# A Lipocalin-Derived Peptide Modulating Fibroblasts and Extracellular Matrix Proteins

**DOI:** 10.1155/2012/325250

**Published:** 2012-04-26

**Authors:** Linda Christian Carrijo-Carvalho, Durvanei A. Maria, Janaina S. Ventura, Kátia L. P. Morais, Robson L. Melo, Consuelo Junqueira Rodrigues, Ana Marisa Chudzinski-Tavassi

**Affiliations:** ^1^Laboratory of Biochemistry and Biophysics, Butantan Institute, Avenida Vital Brasil 1500, 05503-900 São Paulo, SP, Brazil; ^2^Center for Applied Toxinology, Butantan Institute, 05503-900 São Paulo, SP, Brazil; ^3^Department of Orthopedics and Traumatology, Faculty of Medicine, University of São Paulo, 01246-903 São Paulo, SP, Brazil

## Abstract

Lipocalin family members have been implicated in development, regeneration, and pathological processes, but their roles are unclear. Interestingly, these proteins are found abundant in the venom of the *Lonomia obliqua* caterpillar. Lipocalins are *β*-barrel proteins, which have three conserved motifs in their amino acid sequence. One of these motifs was shown to be a sequence signature involved in cell modulation. The aim of this study is to investigate the effects of a synthetic peptide comprising the lipocalin sequence motif in fibroblasts. This peptide suppressed caspase 3 activity and upregulated Bcl-2 and Ki-67, but did not interfere with GPCR calcium mobilization. Fibroblast responses also involved increased expression of proinflammatory mediators. Increase of extracellular matrix proteins, such as collagen, fibronectin, and tenascin, was observed. Increase in collagen content was also observed in vivo. Results indicate that modulation effects displayed by lipocalins through this sequence motif involve cell survival, extracellular matrix remodeling, and cytokine signaling. Such effects can be related to the lipocalin roles in disease, development, and tissue repair.

## 1. Introduction

Development and regeneration are processes driven by dynamic regulation of extracellular matrix (ECM). ECM is continuously exposed to physical and chemical injuries, and its composing proteins are continuously synthesized and secreted by fibroblasts, which play a central role in regulation of tissue homeostasis. Thus, in young and healthy tissue there is a balance between ECM deposition and degradation, in a well-organized and regulated process [1, 2].

Dysfunctions in deposition and remodeling of ECM proteins hinder normal tissue repair and are observed in several pathologies, such as chronic wound [3], sclerosis and other fibrotic diseases [4, 5], tendinopathy [6], diabetes [7], renal disease [8], pulmonary disorders [9], and even heart disease [10]. Many of these have involvement of cytokines. In addition, other undesired conditions such as chrono- and photoaging are associated with breakdown and impaired synthesis of ECM proteins, especially collagen [2].

Interestingly, lipocalin levels are particularly elevated in some of these and other pathological states [11–15], as well as in site-specific injuries [16–18]. Furthermore, expression of lipocalins has been associated with regeneration and tissue repair [16–20], metamorphosis [16, 21, 22], pregnancy [23], chondrogenesis [24, 25], and other processes related to embryogenesis and postnatal development [14, 24, 26–28]. These findings suggest those proteins play a special role in morphogenesis. Lipocalin roles may be beyond their particular lipophilic ligand-binding properties, given the broad phylogenetic range and tissue distribution of lipocalins reported in these studies.

Lipocalins are among the most abundant proteins found in the venom of the *Lonomia obliqua *caterpillar [29, 30]. They are multifunctional proteins with a *β*-barrel structure, which share three characteristic conserved domains in their primary structure, namely, motifs 1–3 [31, 32]. The involvement of motif 2 in cell modulation displayed by lipocalins has been previously demonstrated through a peptide mapping approach studying a toxin from *L. obliqua* [33]. In this study, we investigated the effects of a peptide based on this lipocalin motif on human fibroblasts, evaluating the extracellular matrix proteins in vitro and in vivo, mobilization of intracellular calcium, and mediators involved in cell response.

## 2. Materials and Methods

### 2.1. Reagents and Antibodies

Ham's F-12 culture medium was purchased from Gibco BRL (Grand Island, NY, USA). Fetal bovine serum (FBS) and trypsin-EDTA were from Cultilab (Campinas, SP, Brazil). Vectashield mounting medium was from Vector Laboratories (Burlingame, CA, USA). Monoclonal antibodies to cellular fibronectin, human tenascin, and heat shock protein 47 (HSP47) were from Sigma-Aldrich (St. Louis, MO, USA). Other mouse IgG antibodies were from Santa Cruz Biotechnologies (Santa Cruz, CA). Alexa Fluor 488 goat anti-mouse IgG antibody was from Molecular Probes (Eugene, OR, USA). FLIPR Calcium 4 Assay Kit was obtained from Molecular Devices (Sunnyvale, CA, USA). All other reagents were supplied by Sigma-Aldrich (St. Louis, MO, USA).

### 2.2. Peptide Synthesis

Lipocalin motif-2-derived peptide (pm2b) [33], with the amino acid sequence YAIGYSCKDYK-OH, was obtained in automated benchtop simultaneous multiple solid-phase synthesizer, PSSM 8 system (Shimadzu, Kyoto, Japan) using Fmoc solid phase. The peptide was purified by reversed-phase chromatography semipreparative HPLC (Shim-pack Prep-ODS, Shimadzu), and its purity and identity were confirmed by LC-MS mass spectrometry Surveyor MSQ Plus, Thermo Fisher Scientific (San Jose, CA, USA) and by analytical HPLC.

### 2.3. Cell Culture

Cultures of primary human fibroblasts were obtained from skin biopsies. Cells were grown in Ham's F-12 medium, supplemented with FBS (15%), ampicillin (20 mg/mL), streptomycin (20 mg/mL), and gentamicin (40 mg/mL), at 37°C in a humidified 5% CO_2_ incubator. Having reached confluence, cells were washed three times with phosphate-buffered saline (PBS), detached by mild treatment with trypsin-EDTA, and washed with FBS-supplemented medium. Experiments were carried out with cells from the second passage. To evaluate ECM proteins, fibroblasts (1.15 × 10^5^ cells) were cultured on round slides in 24-well plates incubated with pm2b for 96 h in 500 *μ*L of medium. To obtain cell lysates, fibroblasts were cultured in 25 cm^2^ culture flasks and incubated with pm2b for 96 h. For flow cytometry analysis, fibroblasts (1.5 × 10^5^ cells/mL) were incubated for 72 h with pm2b in 10% or 1% FBS-supplemented medium. All experiments, unless indicated, were carried out with the peptide at 230 nM. Peptide vehicle (150 mM NaCl) was used as control of treatments.

### 2.4. Immunocytochemical Staining for ECM Proteins

Cells were gently washed with PBS and fixed for 15 min in 3% paraformaldehyde, 0.2% glutaraldehyde, 0.1 M phosphate buffer, pH 7.4. Then, slides were washed with PBS and subjected to immunostaining with monoclonal antibody anticellular fibronectin, anti-HSP47, or anti-human tenascin and secondary antibody Alexa Fluor 488, according to manufacturer's instructions. Cells were washed and slides mounted with Vectashield. Slides were visualized under fluorescence microscopy (Carl Zeiss, Jena, Germany) by 200x magnification, and ten microscopic fields were analysed. Expressions of procollagen, fibronectin, and tenascin were quantified through morphometric analysis and digital densitometry using an Image System Analyzer (Kontron Electronic 300, Zeiss). Values were normalized to untreated controls.

### 2.5. Western Blotting

Cells were lysed in RIPA buffer for 20 min at 4°C and centrifuged for 5 min at 21,000 g, to obtain soluble and insoluble extracts. The cell lysate of treated (77 and 230 nM pm2b) and control fibroblasts were subjected to SDS-PAGE, using 30 *μ*g of each protein sample of soluble extracts. Fibronectin and laminin were analyzed using the insoluble extracts. Then, proteins were electrotransferred to nitrocellulose membrane, which was blocked with 1% bovine serum albumin in 20 mM Tris-HCl pH 7.4, 0.15 M NaCl, and 0.05% Tween (TBS-T) and incubated in the same buffer with primary antibodies for fibronectin, laminin, collagen type I, HSP47, and GADH as control. Membranes were washed with TBS-T and incubated with secondary antibody conjugated with alkaline phosphatase. Incubation with each antibody was according to manufacturer's instructions. Nitro blue tetrazolium and 5-bromo-4-chloro-3-indolylphosphate (NBT/BCIP) substrates were used for immunostaining. Proteins were quantified through digital densitometry using the ImageJ software (National Institutes of Health, USA). Values were normalized to untreated controls.

### 2.6. In Vivo Treatment

BALB/c mice (20 ± 2 g) were bred at Butantan Institute. Animals had free access to food and water and were in a light-dark cycle of 12 h. Mice were anesthetized with ketamine (75 mg/kg) and xylazine (10 mg/kg) i.p. and had the dorsum shaved. The animals were divided into 2 groups, treated with intradermal injections of a single dose of pm2b (0.2 mL, 1.15 *μ*M, *n* = 6) or two repeated doses (7 days of interval, *n* = 4) in a delimited site on the dorsum. Paired controls were injected with the vehicle (saline) in a delimited site on the opposite side in the same animal. Skin fragments of 1 × 1 cm from each site (pm2b-treated and control) were collected in pairs of treated mice in intervals of one, two, and twelve weeks after single treatment, as well as one and twelve weeks after the first dose of repeated treatment. Skin samples were immediately fixed in 10% buffered-formalin for histological procedures. Mice were euthanized prior to biopsies. All procedures were performed in compliance with the tenets of the Brazilian Society of Laboratory Animal Science (SBCAL/COBEA) and the institutional ethics committee.

### 2.7. Histological Analysis

Tissue sections of 3 *μ*m thickness were stained with picrosirius red and examined under light microscopy (Carl Zeiss, Jena, Germany) coupled to Kontron 300 System Image Analyzer. Quantitative analysis of collagen was done from 10 microscopic fields at 200x magnification on flat sections of each biopsy through morphometric analysis and digital densitometry. Data were expressed as percentage of collagen staining to total area and in function of matched controls.

### 2.8. Calcium Mobilization Assay

Changes in free intracellular calcium concentration were measured by microfluorimetry using the FlexStation 3 (Molecular Devices, Sunnyvale, CA, USA) and FlexStation Calcium Assay Kit, following manufacturer's instructions. Fibroblasts were seeded at a density of 5 × 10^4^ cells per well in black-well plates with clear bottom. Prior to experiments, cells were incubated for 1 h, at 37°C with the calcium kit reagent in serum-free medium. Before measurements, the following treatments were added: ATP (10 *μ*M), thapsigargin (1 *μ*M), and pm2b (80 or 230 nM, immediately and 1 h before). Both the direct effect of pm2b and also its influence on calcium mobilization by thapsigargin were investigated. The inhibitory effect of BAPTA (10 *μ*M, 30 min before) was used as a positive interference control. Fluorescence was measured during 120 s, at 1.52 time intervals. Measurements were obtained as the difference between the peak intensity fluorescence and baseline.

### 2.9. Flow Cytometry Analysis

Cells were gently washed with PBS and detached with trypsin-EDTA. After addition of FBS 10%, the cells were harvested and washed twice with PBS. Pellets were resuspended in 4% paraformaldehyde and stored at 4°C. Antibody labeling was done according to manufacturer's instructions at room temperature. Prior to analysis, cells were permeated with 0.1% Triton X-100 for 30 min, incubated for 2 h with the respective antibodies for caspase 3, Bcl-2, Ki-67- MIB-1, cytochrome c (cyt c), IL-1*β*, CXCR1, CXCR2, IL-6R, or collagen-1 receptor (*α*2*β*1), and then incubated with the secondary antibody Alexa Fluor 488 in the dark. Fluorescence-activated cell sorting (FACS) analysis was performed on a FACSCalibur flow cytometer, Becton Dickinson (San Jose, CA, USA). For each sample, at least 10,000 events were acquired and the data were evaluated using the Cell-Quest software.

### 2.10. Statistical Analysis

The difference among groups was analyzed by one-way or two-way analysis of variance (ANOVA) and Student's *t*-test. Data are expressed as mean ± standard error (SEM) and representative images from each group. Differences were considered statistically significant when *P* < 0.05.

## 3. Results

### 3.1. Increased Production of ECM Proteins in Fibroblast Culture

To evaluate if pm2b was able to modulate fibroblast response, we assessed if the peptide could interfere with the production of ECM proteins by primary human fibroblasts in culture.

**Figure 1:**
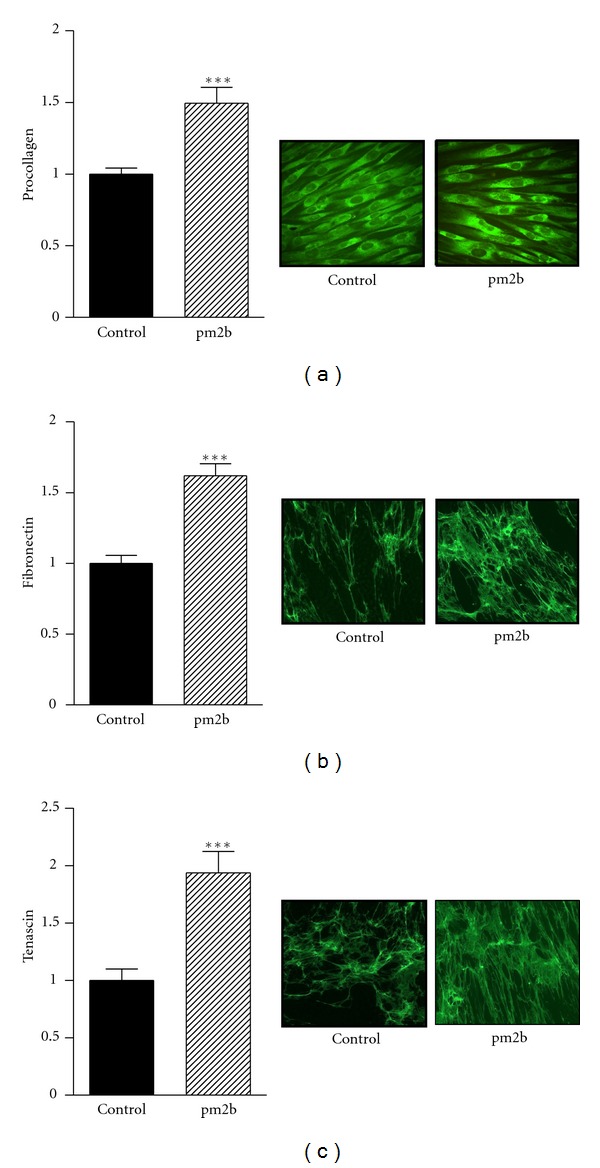
Extracellular matrix proteins in fibroblast culture. Procollagen (a), cellular fibronectin (b), and tenascin (c). Primary human fibroblasts were treated with pm2b (230 nM) and proteins were analyzed after 96 h by immunocytochemical staining (originally 400x). Data are representative images and expressed as mean ± SEM for triplicate measurements. ***P < 0.001 versus control.

**Figure 2:**
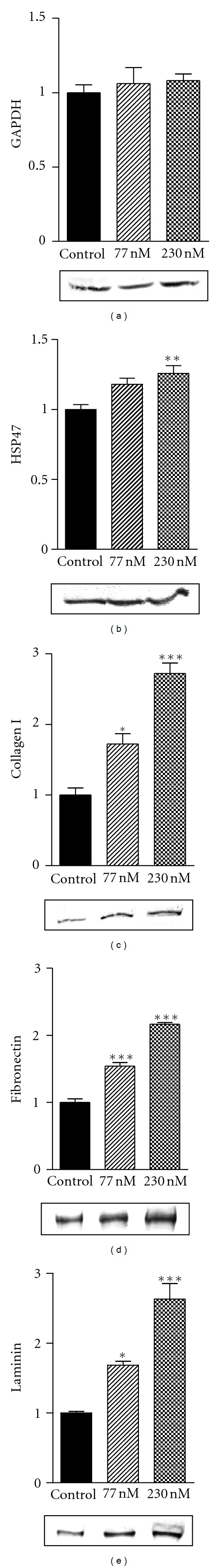
Extracellular matrix proteins in fibroblast lysate. Primary human fibroblasts were treated with pm2b (77 or 230 nM) and proteins were analyzed after 96 h by western blotting. Data are representative images and expressed as mean ± SEM for duplicate measurements. ***P < 0.001, **P < 0.01, *P < 0.05 versus control.

Immunofluorescence results showed that treatment with pm2b induced a significant increase of procollagen, fibronectin, and tenascin, as shown in Figure 1. In comparison to nontreated cultures, pm2b-treated fibroblasts showed almost onefold increase in tenascin (94%) and an increase in 49% of procollagen and 62% of fibronectin. Analysis of collagen type I, fibronectin, and laminin in the cell lysates by western blotting showed a significant increase in all these proteins in cultures treated with the peptide at 70 or 230 nM and a slight increase in HSP47 (Figure 2).

### 3.2. Increased Production of Collagen In Vivo

**Figure 3:**
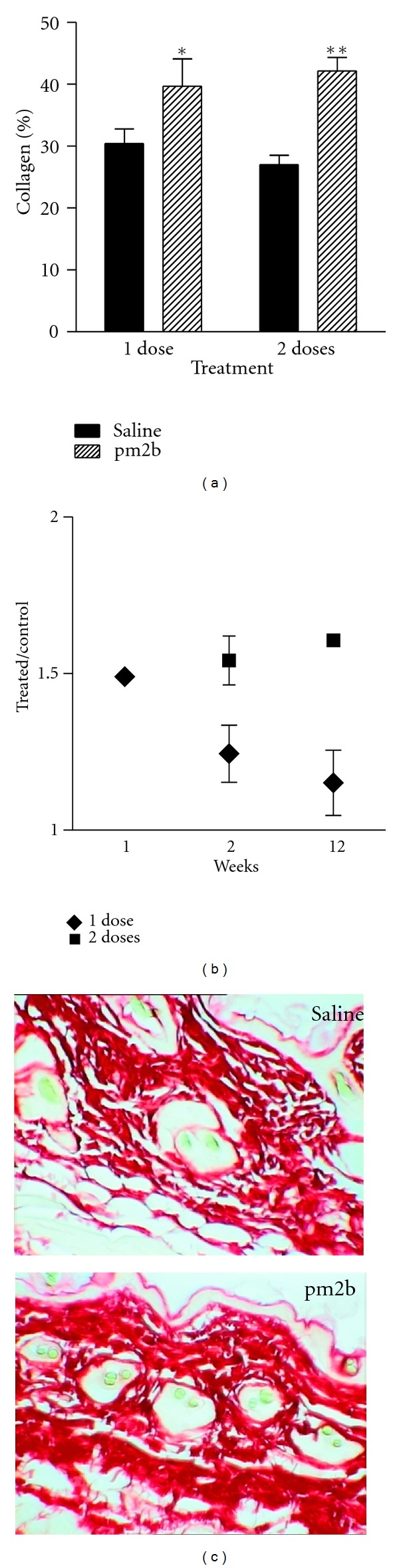
Peptide treatment increases collagen in the mice dermis. (a) Mean values of groups treated with a single dose (0.2 mL, 1.15 *μ*M pm2b, i.d.) or repeated doses compared with controls (*n* = 4–6). (b) Matched observations of pm2b-treated and saline-treated sites (1 × 1 cm) in the same animals in successive intervals after treatment. (c) Picrosirius-red-stained sections (originally 340x). Data are representative images and expressed as mean ± SEM. **P < 0.01, *P < 0.05 versus control.

Since the peptide treatment induced a change in the content of ECM proteins in vitro, we assessed whether if pm2b was also able to increase the amount of collagen in vivo in the mice dermis (Figure 3). Interaction of pm2b treatment and the collagen content was statistically significant (two-way ANOVA, *P* < 0.05), either if it was lower than that observed in vitro. Treatment with a single dose induced a mean increase of local collagen fibrils in about 10%, while with two repeated doses the mean increase was 15% (Figure 3(a)). With a single dose, the higher difference to controls was observed 7 days after treatment. The ratio between treated area and control dropped along the time. Interestingly, in the group treated with 2 doses of pm2b, the collagen increase lasted for 3 months (Figure 3(b)).

### 3.3. Calcium Mobilization

**Figure 4:**
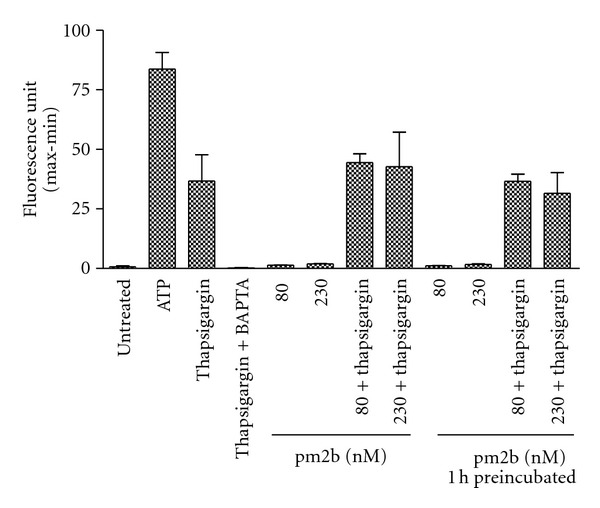
Calcium mobilization in fibroblasts. Fibroblasts were treated with pm2b at different concentrations immediately and 1 h before the test, and with thapsigargin (1 *μ*M), BAPTA (10 *μ*M, 30 min before), and ATP (10 *μ*M). Data are expressed as mean ± SEM for duplicate measurements.

Increase of intracellular calcium in fibroblasts was observed by using ATP or thapsigargin (Figure 4). As expected, pretreatment with BAPTA abolished the effect of thapsigargin. On the other hand, the peptide investigated showed no direct effect on intracellular calcium mobilization. In addition, it did not seem to interfere with the action of thapsigargin.

### 3.4. Modulation of Mediators Involved in Cell Viability and Inflammation

**Figure 5:**
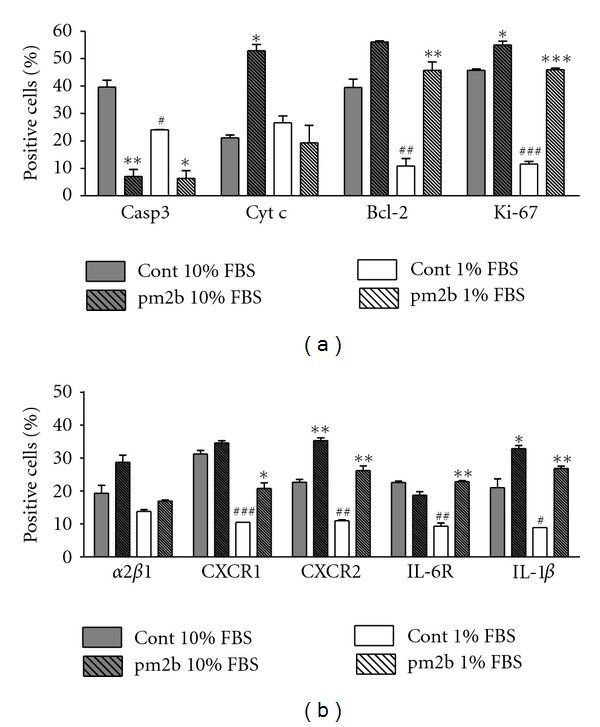
Differential expression of mediators involved in cell viability (a), cytokine and receptors (b). Fibroblasts were cultured with pm2b (230 nM) for 72 h with 10% or 1% FBS and analyzed by FACS. Data are expressed as mean ± SEM for triplicate measurements. ***P < 0.01, **P < 0.01, *P < 0.05 versus controls. ^###^ *P* < 0.01, ^##^ *P* < 0.01, ^#^ *P* < 0.05 versus controls with 10% FBS.

To investigate the mechanisms underlying fibroblast responses induced by pm2b, a set of mediators expressed by the cells in 1% and 10% FBS-supplemented medium were analyzed. Either treated or nontreated cultures showed different responses in these two conditions. pm2b promoted a synergistic modulation of mediators involved in apoptosis, antiapoptosis, and proliferation, resulting in a prosurvival response. As seen in Figure 5(a), pm2b suppressed caspase-3 and upregulated Bcl-2 and Ki-67. As shown in Figure 5(b), increases in CXCR1 and IL-6R were observed only with 1% FBS. On the other hand, cyt c was exclusively upregulated in 10% FBS-supplemented medium. In both conditions, there was a slight increase in the *α*2*β*1expression, but not statistically significant. CXCR2 and IL-1*β* were markedly increased in both culture conditions.

## 4. Discussion

It is well known that morphogenesis and other physiological processes consist in a chain of events regulated by cross-talk signaling between ECM cells, receptors, and signaling factors [34]. However, lipocalin roles in these processes are not clearly understood. Regardless of many reports describing lipocalins as biomarkers of diseases [11] and others that correlate high lipocalin expression levels with stress conditions [35] and injury [16, 17], little is known about the biological activities of these multifunctional proteins and how they can modulate tissue and cell responses.

Lipocalins are classically recognized as carriers of lipophilic molecules. However, outgrowing data on the literature have indicated they are more than that. Some authors have suggested lipocalin motifs should play important roles in the structure pattern and functional properties of these proteins [31, 32, 36]. Bioinformatic analysis and peptide mapping indicated motif 2 is implicated in cell modulation and suggested it is a sequence signature with a role in cell survival [33]. However, its possible effects in fibroblasts were not known.

We have obtained a synthetic peptide with amino acid sequence based on the lipocalin motif 2, found in Lopap—an insect lipocalin from the *L. obliqua *caterpillar [37]. Lopap was previously shown to have a direct effect on endothelial cells increasing the surface expression of cell adhesion molecules, triggering IL-8 and nitric oxide release, and displaying antiapoptotic activity [36–40]. The peptide reproduced the effects observed with the whole protein, exhibiting an antiapoptotic activity in endothelial cells and neutrophils, which is dependent on nitric oxide synthase activity [33].

Herein, results demonstrate the peptide can modulate mediators favoring a prosurvival response, with suppression of caspase 3—a key proapoptotic enzyme, and up-regulation of the antiapoptotic protein Bcl-2, as well as the proliferation marker Ki-67. Other studies have also attributed to lipocalins roles in cell survival in different cell lineages [41–43], which support the hypothesis of a common property among lipocalins. This effect can be important for the lipocalin roles in several developmental and repairing processes, for protective response to stress, as well as for their possible involvement in many diseases.

Interestingly, pm2b treatment increased ECM proteins in vitro and in vivo. Fibroblasts are metabolically active cells which major function is the production of ECM components [1]. Our findings suggest for the first time a lipocalin role in ECM modulation. This finding brings new insights to understanding the involvement of lipocalins in the pathophysiology of diseases involving ECM deposition/remodeling defects. The difference in the amount of collagen increase induced by pm2b in vitro and in vivo may be due to collagen degradation by matrix metalloproteinases or either the peptide dose used and its stability in the tissue. Ki-67 is absent in resting cells and present during all phases of cell cycle, but otherwise its role in ribosomal RNA synthesis [44] may be related to the increase in synthesis activity observed in fibroblasts.

Fibroblast modulation by pm2b was shown to involve cytokine signaling favoring a proinflammatory response. Besides the previously reported modulation of IL-8 by Lopap [39], results showed the induction of the IL-8 receptors. Expression of the chemokine receptor CXCR1 was increased only in serum-deprived cultures, while expression of its paralog CXCR2 was increased no matter the assay condition. IL-8 and its receptors are known to have an autocrine role in cell survival [45]. There are also reports describing the involvement of IL-1 [46] and IL-6/IL-6R [47] in antiapoptotic responses. On the other hand, a local proinflammatory response can contribute for healing and regeneration [48]. However, the mechanisms by which Lopap and derived peptide trigger cell responses have to be investigated. As results show, it does not seem to involve changes in calcium transients.

To our knowledge, this is the first report of lipocalins modulating fibroblasts and ECM proteins, which could be directly implicated to their roles in morphogenesis and tissue homeostasis. The involvement of growth factors and cytokines in development and repairing process is well described [49]. However, lipocalins may be also considered as important players in these processes. Therefore, the mechanisms by which these proteins can trigger cell modulation have to be carefully investigated. Understanding the effects of these proteins can open perspectives for their use in prognosis and treatment of many dysfunctions involving wound healing, tissue remodeling, and cell death.
